# PreView: Development and Pilot Testing of an Interactive Video Doctor Plus Provider Alert to Increase Cancer Screening

**DOI:** 10.5402/2013/935487

**Published:** 2012-12-06

**Authors:** Millie Arora, Barbara Gerbert, Michael B. Potter, Ginny Gildengorin, Judith M. E. Walsh

**Affiliations:** ^1^Division of General Internal Medicine, Department of Medicine, University of California San Francisco, San Francisco, CA 94143, USA; ^2^Division of Behavioral Sciences, Professionalism and Ethics, University of California San Francisco, San Francisco, CA 94143, USA; ^3^Department of Family and Community Medicine, University of California San Francisco, San Francisco, CA 94143, USA

## Abstract

*Background*. Interventions to increase recommended cancer screening tests and discussions are needed. *Methods*. We developed the PREventive VIdeo Education in Waiting Rooms Program (PreView), a multimedia cancer prevention intervention for primary care clinics based on the Transtheoretical Model of Behavior Change. We pilot tested PreView, an interactive Video Doctor plus Provider Alert for feasibility and acceptability in primary care clinic settings in the San Francisco Bay Area , CA in 2009-2010. *Results*. Eighty participants (33 men and 47 women; more than half non-White) at 5 primary care clinics were included. After PreView, 87% of women were definitely interested in mammography when due, and 77% were definitely interested in a Pap test. 73% of participants were definitely interested in colorectal cancer screening when due, and 79% of men were definitely interested in a discussion about the PSA test. The majority indicated that they received an appropriate amount of information from PreView and that the information presented helped them decide whether or not to be screened. *Conclusions*. PreView was well received and accepted and potentially provides an innovative and practical way to support physicians' efforts to increase cancer screening.

## 1. Background

Cancer screening saves lives and is universally recommended for several cancers, including breast, cervical, and colorectal cancers. For women ages 50 and over, breast, cervical, and colorectal cancer screening are recommended. For men in the same age range, recommended screening includes colorectal cancer screening, and, due to controversy surrounding the benefits and risks of prostate cancer screening, shared decision making is recommended [1–4]. 

Currently rates of cancer screening are suboptimal. According to the 2010 National Health Interview Survey, 72.4% of women were screened for breast cancer, 83.0% of women reported being screened for cervical cancer in the previous 3 years, and 58.6% of adults reported being up to date with colorectal cancer screening [5]. Although many men are screened with PSA, it is not known how many of them do so after a shared decision-making discussion. There are many reasons people may not be receiving screening, including patient factors (e.g., not thinking it is important to get screened, concerns about discomfort), provider factors (e.g., time demands, the need to discuss more urgent issues, uncertainty about recommendations), and system factors (e.g., access to care) [6]. 

Multimedia interventions have been shown to be feasible and acceptable in providing information and increasing knowledge of newly diagnosed patients with prostate cancer [7]. Multimedia interventions have been shown to be effective in increasing knowledge about breast cancer and intention to ask the provider about screening in Latinas [8]. In addition, 40% of women who obtained or scheduled a mammogram attributed their decision to the intervention [9]. Multimedia and other interventions designed to increase rates of cancer screening typically addresses one type of cancer. Our intervention is unique in that it focuses on increasing rates of screening and screening discussions for all appropriate cancers for a given individual.

We have developed and successfully tested our innovative, interactive, multimedia “Video Doctor” to assess patients' sexual risks, as well as to reduce drug use, smoking, risky sexual behaviors, and to increase exercise and healthy eating [10–16]. The Video Doctor interacts with the patient and then encourages the patient to continue discussion with his or her real physician. In our previous work, participants in the intervention group were significantly more likely to report provider-patient discussions of intimate partner violence compared with participants receiving usual care at baseline (81.8% versus 16.7%; *P* < .001) [17]. When the patient completes the Video Doctor interaction, the program produces a Provider Alert for use during the medical appointment, offering the real physician an at-a-glance summary of the patient's risk profile and readiness to change, as well as suggested counseling statements. When specifically asked about the helpfulness of these discussions in a previous study, 20 out of 22 (90.9%) participants rated the discussion as helpful or very helpful at baseline [17]. Although the Video Doctor has been proven successful in reducing smoking, risky sexual behavior, in increasing exercise and healthy eating, and increasing provider-patient discussions of domestic violence, it had not previously been used or evaluated to encourage recommended cancer screening. 

The aim of this study was to develop and pilot test PreView, a novel interactive Video Doctor plus Provider Alert, that can be implemented during a primary care visit to encourage all cancer screening and cancer screening discussions that are recommended for a particular individual.

## 2. Methods

### 2.1. Development and Content of the Intervention

#### 2.1.1. Overview

We developed PreView (the PREventive VIdeo Education in Waiting Rooms Program). This Video Doctor plus Provider Alert program is a multimedia tool that utilizes a touch screen computer with an interactive interface in which participants answer questions about demographics, health history, prior cancer screening, screening stage of change (based on the Transtheoretical Model of Behavior Change [18], and perceived barriers to screening and receive messages about their breast, cervical, colorectal and prostate cancer screening behavior. Participants use the Video Doctor just before their appointment with their physician. The Video Doctor (1) assesses eligibility for cancer screening or cancer screening discussions, (2) guides the participant through a series of assessment questions, and (3) interacts with the participant and provides individualized messages based on their stage of change and individual screening barriers for each cancer and (4) generates a Provider Alert that is designed to assist the primary care physician in providing the right information and screening tests for each participant. 

#### 2.1.2. Assessment

PreView starts with a welcome to the Video Doctor Program and a series of health and risk assessment questions, asking about prior cancer screening (whether or not a person has ever been screened or is up-to-date with screening) and readiness to change cancer-screening behavior. Women are asked about breast, cervical, and colorectal cancer screening tests and men are asked about colorectal cancer screening tests and whether they have ever had a discussion about prostate cancer screening with their provider. The stages of change are based on the Transtheoretical Model (TTM) and are assessed for each cancer [18, 19]. For example, for breast cancer screening, the description for each stage would be as follows: (1) Precontemplation: has no intention to have a mammogram in the next 6 months, (2) Contemplation: intends to have a mammogram in the next 6 months, (3) Action: has had at least one mammogram in the past and taken some behavioral steps in this direction, (4) Maintenance: has had mammograms in the recommended time interval, and (5) Relapse: has had a mammogram in the past, but does not intend to have another screening test. For prostate cancer, the focus is on the discussion of the screening test rather than on receipt of the test.

#### 2.1.3. The Video Doctor

The Video Doctor is introduced and is designed to simulate a conversation between patient and a real physician, including video and audio in order to give the participant a realistic experience. The program uses branching logic in order for individually relevant and appropriate video clips to be shown according to the participant's previous answers.

The participant receives an individualized message from the Video Doctor based on his/her stage of change for each individual cancer. For example, for breast cancer, individuals who are Precontemplators receive a message about the importance of routine mammography, whereas those in Maintenance receive a congratulatory message for taking care of their health. Participants then receive information about norms and recommendations for screening for each cancer. Next, individuals are asked about their readiness to change for each cancer. Based on their response, the participant receives an individualized message about the relevance, risks, and rewards of changing. Participants are defined as not ready to change (Precontemplation or Relapse), unsure about change (Contemplators), and ready to change (Action). Individuals who are not ready or unsure receive *individualized* messages from the Video Doctor about the relevance, risks, and rewards of screening and then choose from a list of potential barriers, or roadblocks to screening. 

#### 2.1.4. Roadblocks

Each individual can choose up to 3 roadblocks per cancer, and later receives responses tailored to each individual roadblock, including suggestions about how to overcome each roadblock. Roadblocks differ for each cancer and they are based on our previous work and the work of others in determining and overcoming barriers to colorectal, breast and cervical cancer screening [20–23]. For prostate cancer shared decision making, barriers were developed based on published shared decision making research [24, 25]. We identified 9 roadblocks for mammography, 8 roadblocks for Pap tests, 15 roadblocks for colorectal cancer screening and 12 roadblocks for PSA discussion. After all the roadblocks chosen by an individual (up to 3) have been addressed, individuals are encouraged to discuss any concerns they may have with their real doctors. Roadblocks for each cancer are described in Table 1.

#### 2.1.5. Provider Alerts

For each participant, the program then produces a *Provider Alert* for each cancer, a printed sheet summarizing the individual patient's screening history, his/her individual readiness to change, and his/her individual roadblocks and suggestions to the physician for appropriate roadblock-related messages. The *Provider Alert* is given to the physician with the patient's chart at the visit. The physician can discuss the patient's relevant concerns, then check a series of boxes about what happened at today's visit, indicating what he/she asked the patient to do, and can then sign it and return part of the sheet to the patient where it serves as a “prescription for prevention.”

#### 2.1.6. Postvisit Assessment

After the physician visit, the participant returns to the computer for a postvisit assessment, which again assesses intention to change screening behavior, asks whether each cancer screening test was ordered or discussed during the visit. Men are asked if they engaged in shared decision making about prostate cancer screening during the visit.

## 3. Pilot Study

### 3.1. Usability Testing

#### 3.1.1. Settings

The pilot testing was conducted at five primary care clinics. One other site additionally agreed to participate but then dropped out due to competing priorities in the office at the time of the study. All clinics were participants in the San Francisco Bay Area Collaborative Research Network (SF Bay CRN), a primary care practice-based research network affiliated with UCSF's Clinical and Translational Sciences Institute. Two sites were community-based primary care clinics at the University of California San Francisco (UCSF), and three sites were smaller independently run community-based primary care clinics. These clinics were recruited and selected in order to provide a range of practice settings with ethnically and socioeconomically diverse patients in which to test the intervention for feasibility and acceptability.

#### 3.1.2. Patient Participants

Eligibility criteria included: (1) being between the ages of 50 to 70; (2) no prior history of cancer; and (3) ability to speak English. Potential participants were identified from a clinic-generated list of potentially eligible men and women who had upcoming appointments to see their primary care physicians. Physicians approved the contact of all patients; potential participants were then recruited and screened by telephone. 

#### 3.1.3. Intervention

Participants arrived one hour prior to their scheduled appointment to enroll in the study. After completion of an informed consent, participants used a touch screen computer in the clinic waiting room to access the Video Doctor Plus Provider Alert program. The program was self-administered with a research assistant on site if the participant needed assistance. Headphones were used to ensure privacy. After the participant completed the program, which included interaction with the Video Doctor, a Provider Alert was generated for the provider. The Provider Alert summarized the individual's stage of change and perceived barriers to screening for each cancer. The goal of the Provider Alert was for the real physician to discuss each patient's individual stage of change (based on the Transtheoretical Model of Behavior Change) and barriers at the visit. The provider could also tear off a “prescription for change” at the bottom of the Provider Alert and give it to the patient. After visiting with the doctor, the patient participant completed a post-visit questionnaire.

#### 3.1.4. Measures and Outcomes

*Measures*. The program asked about demographic information including age, gender, race/ethnicity, language, education level, marital status, employment status, health insurance, self-rated health, preferred method of health decision making, and if women ever had a hysterectomy. The program also asked about the participant's stages of change, screening history, and perceived barriers to screening.

After the visit, we asked the participant about the intention to ask the provider about screening and whether or not the participant was interested in being screened, whether he or she received the right amount of information from PreView, whether or not PreView increased knowledge, whether or not PreView helped him/her decide to be screened or not, and readiness to have the test or discussion. 

For the pilot study, the primary outcome measures were: (1) the participant's intent to ask the provider about screening and (2) the participant's interest in screening after PreView. All study procedures were approved by the UCSF Institutional Review Board. Data were collected from April 2009 through August 2010.

Data were analyzed with the statistical software package, SAS version 9.2 [26]. We used descriptive statistics including means, standard deviations and percentages to assess the demographic characteristics of the group and to evaluate participant responses after the intervention. 

## 4. Results

### 4.1. Usability Testing

Usability testing was conducted to ensure that participants were able to understand how to use the program and to ensure that the branching flowed smoothly. A total of 24 individuals participated in the usability testing phase. Participants spent 30 minutes on average with PreView. All 24 participants completed the colorectal cancer screening module. All of the women completed both the breast and the cervical cancer modules. The four men all completed the PSA discussion module. There was no predominant “most preferred” or “least preferred” module. Based on the results of the usability testing, some modifications were made to the branching logic to ensure that the messages were appropriate and the branching patterns were correct. All liked the touch screen computer and all found the computer easy to use, even those who did not know how to use a computer. 

### 4.2. Pilot Testing Results

#### 4.2.1. Participant Characteristics

Participant characteristics are described in Table 2. Eighty individuals participated in the pilot phase of the study. There were 33 men and 47 women. Slightly more than half were non-White, and more than two-thirds had a college degree or higher. The majority had some type of medical insurance. 79% of participants rated their health as good, very good, or excellent. Slightly less than half stated they preferred to make health decisions with their doctor, and about 40% liked to make the decision themselves after listening to their doctor's opinion. With the exception of one participant who was unable to read, once the PreView program started, no other participants required additional assistance from the research assistant in navigating PreView. 

#### 4.2.2. Responses of Study Participants after PreView

Participants completed PreView before their doctor's visit. After visiting with their doctor, participants answered questions about their intentions to be screened for breast, cervical, or colorectal cancer or to discuss prostate screening. Responses are described in Table 3. 87% of women were definitely interested in receiving a mammogram and 77% of women said they were definitely interested in receiving a Pap test when they were next due. Almost two-thirds of participants were definitely interested in receiving a colorectal cancer screening test the next time they were due, and 79% of men would definitely be interested in a discussion about PSA testing with their physicians. Additionally, 85% of women said they would be very likely to ask their doctor about a mammogram when it was next due, and 73% would be likely to ask about Pap tests when due. 85% of all participants said they would be somewhat or very likely to ask their doctors about a colorectal cancer test when it was next due.

For all cancers, the majority of participants said that the amount of information they received from PreView was about right. Additionally, about 70% of all participants agreed or strongly agreed that the information provided in the Video Doctor program helped them decide whether or not to be screened for breast, cervical or colorectal cancer, or whether to have a discussion about prostate cancer screening with their providers. 

## 5. Discussion 

PreView is a novel interactive Video Doctor plus Provider Alert Program designed to be used before a patient's appointment with his or her primary care physician. PreView is based on the Transtheoretical Model of Behavior Change. The goal of PreView is to provide support for the physician and simplify the visit by assessing an individual's stage of change for cancer screening and identifying individual barriers to screening so that the physician can most effectively address a particular patient's barriers to being screened for breast, cervical, or colon cancer or to discuss prostate screening.

After using the PreView Program and interacting with the Video Doctor, most patients were planning to ask their doctor and had interest in screening tests for breast, cervical or colorectal cancer and in discussing prostate cancer screening. In addition, the vast majority of participants felt that PreView provided them with the right amount of information, and that the information presented by PreView helped them decide whether to be screened for each cancer.

Prior studies have shown that computer multimedia tools are effective when targeting a single disease or type of screening. Multimedia tools have been used in the waiting room and have been successful in leading to appropriate intensification of diabetes therapy [27], and have been shown to be feasible and effective in facilitating confidence in treatment choices in patients with newly diagnosed prostate cancer [7]. 

Interactive multimedia waiting room interventions have been evaluated for breast cancer screening and colorectal cancer screening. 1197 Latina women were exposed to an interactive multimedia breast cancer intervention that addressed risk, the importance of early detection, and the role of mammography [8]. Four months after the intervention, 40% of the women who scheduled or obtained mammography attributed their decision to the intervention [9]. In a separate study, Latino men and women viewed a multimedia intervention in the waiting room of community clinics. At posttest participants were significantly more likely to consider colorectal cancer screening options and to discuss screening with their physician [28]. PreView is unique because it does not focus on only one cancer, but rather it addresses *all* cancer screening that is recommended for a given individual aged 50 or older, and we have now shown that it is feasible and effective for use in primary care practice.

The Video Doctor plus Provider Alert is an innovative interactive, multimedia program that has previously been shown to be successful in reducing a multiplicity of risky behaviors and increasing a number of health promoting behaviors. It has been effective in encouraging patients to reduce their smoking and drug use, and their risky sexual behaviors. It has also been successful in encouraging an increase in exercise and healthy eating. Further, the Video Doctor plus Provider Alert has increased provider-patient discussions of domestic violence for patients with a history of such violence, and, when these discussions occur, patients report that they value talking with their provider about this sensitive topic [10–17]. 

PreView extends the Video Doctor plus Provider Alert model into additional prevention arenas. PreView is unique in that it addresses several types of cancer screening, whereas previous multimedia interventions have only addressed one type of cancer screening [8, 9]. Since physicians routinely address several types of cancer screening with an individual patient, PreView has great clinical relevance and potential.

A limitation of the study is that we did not measure cancer screening outcomes. However, the goals of this study were to develop, pilot test, and assess the feasibility and acceptability of PreView in the primary care setting while addressing multiple cancers. In addition, although this was a pilot study, it was done in diverse clinical settings, reflective of the real world of primary care. 

## 6. Conclusion

PreView augments the patient-provider interaction by directly addressing any barriers a particular patient may have to being screened for various cancers, without requiring any additional time or effort from the physician. We have shown that PreView is feasible and acceptable for use in primary care settings, and that the majority of patients plan to discuss cancer screening with their physicians after interacting with the program. Based on the successful results of this pilot study, we are currently evaluating the impact of PreView on cancer screening rates and cancer screening discussions in a randomized controlled trial. 

Innovative, effective ways to support physicians in cancer screening must be implemented and evaluated. PreView was well received and could provide a practical way to support providers' efforts to increase appropriate cancer screening and cancer screening discussions and would be cost effective in the long term.

## Figures and Tables

**Table 1:** Barriers by cancer type.

	Breast	Cervical	Colorectal	Prostate
Telephone	I have a hard time getting help on the phone	I have a hard time getting help on the phone	I have a hard time getting help on the phone	

MD access	I have a hard time seeing a doctor	I have a hard time seeing a doctor	I have a hard time seeing a doctor to get the test	

Communication with providers/staff	I have questions for the doctor	I have questions for the doctor	I have questions for the doctor	I have questions for the doctor, or I am uncomfortable or embarrassed asking questions I have difficulty interrupting, disagreeing with, or expressing my opinion to the doctor

Perceived risk	I do not think I am at risk for breast cancer	I do not think I am at risk for cervical cancer	I do not think I am at risk for colon cancer	

Perceived need			I do not need to get tested because I do not have any symptoms I do not need it because I have a healthy lifestyle My doctor did a rectal examination so I do not need the test	

Information needs	I need more information about the test	I need more information about the test	I am not sure what test to have	

No MD recommendation	My doctor did not recommend it	My doctor did not recommend it	My doctor did not recommend it	My doctor did not recommend it

Regular “annual” screening is a problem	Getting a mammogram regularly is a problem		Getting a stool test every year is a problem	

Health problems get in way of screening	I have too many other health problems to get tested	I have too many other health problems to get tested	I have too many other health problems to get tested	I have too many other health problems to get tested

Continuity of care	I do not have a regular doctor	I do not have a regular doctor	I do not have a regular doctor	I do not have a regular doctor

Knowledge				Is not all cancer bad? I am worried about the side effects of treatment

Time				I do not want to take up too much of the doctor's time

Medical terms				I have trouble understanding medical terms

Confusion with digital rectal exam				If I had a rectal exam, I do not need a PSA test

MD discussion				My doctor has in the past ordered PSA but did not discuss it

Logistics			I do not have a stool test kit	

Test concerns			I have concerns about sigmoidoscopy or colonoscopy	

**Table 2:** Characteristics of study participants in San Francisco, California in 2009-2010, *N* = 80.

Characteristic	*N* (%)
Number of participants from each clinic	
Clinic 1	19 (24%)
Clinic 2	18 (23%)
Clinic 3	12 (15%)
Clinic 4	10 (13%)
Clinic 5	21 (26%)
Age, y	
Mean ± SD (min, max)	60.6 ± 7.4 (50.0, 86.1)
Gender	
Male	33 (41%)
Female	47 (59%)
Ethnicity	
Asian	7 (9%)
Black or African-American	21 (26%)
White	37 (46%)
Some other race	15 (19%)
Language	
English	69 (86%)
Spanish	5 (6%)
Other	6 (8%)
Education	
High school, GED, or less	28 (35%)
College degree	23 (29%)
Some graduate school or more	29 (36%)
Marital status	
Married/living with domestic partner	46 (58%)
Single/divorced/widowed	34 (43%)
Employment	
Working full time or part time	38 (48%)
Unemployed or looking for work/unable to work for health reasons/fulltime student or homemaker	8 (13%)
Retired	32 (40%)
Health insurance status	
Medicare/Medicare and Medicaid/Medicare and private	33 (42%)
Private insurance	36 (46%)
None	0 (0%)
Not sure or other	10 (13%)
Self-rated health	
Excellent/very good	34 (43%)
Good	29 (36%)
Fair/poor	17 (21%)
Health decisions	
I prefer to make the decision myself	4 (5%)
I prefer to make the decision after I listen to my doctor's opinion	31 (39%)
I prefer that my doctor and I make the decision together	39 (49%)
I prefer that my doctor make the decision after considering my opinion	4 (5%)
I prefer that my doctor make the decision	2 (3%)
For women: have you had a hysterectomy? (*n* = 34)	
Yes	4 (12%)
No	30 (88%)

**Table 3:** Responses of pilot study participants after PreView the Interactive Video Doctor plus Provider Alert in San Francisco, California in 2009-2010, *N* = 80.

Question	Mammogram	Pap test	Colon test	PSA discussion
Did you discuss today?*	(*n* = 34)	(*n* = 31)	(*n* = 62)	(*n* = 28)
Yes	21 (62%)	15 (48%)	50 (81%)	25 (89%)
No	13 (38%)	16 (52%)	12 (19%)	3 (11%)
Interest in test/discussion when it is due?	(*n* = 47)	(*n* = 44)	(*n* = 80)	(*n* = 33)
Definitely	41 (87%)	34 (77%)	58 (73%)	26 (79%)
Somewhat	3 (6%)	3 (7%)	6 (8%)	2 (6%)
Undecided	2 (4%)	1 (2%)	9 (11%)	—
Not very/not at all	1 (2%)	6 (14%)	7 (9%)	5 (15%)
How likely to ask MD about it when it is due?	(*n* = 47)	(*n* = 44)	(*n* = 80)	(*n* = 33)
Very likely	40 (85%)	32 (73%)	59 (74%)	24 (72%)
Somewhat	2 (4%)	3 (7%)	9 (11%)	3 (9%)
Not very/not at all	4 (9%)	9 (21%)	11 (14%)	6 (18%)
No answer	1 (2%)	—	1 (1%)	—
Amount of information received was?	(*n* = 47)	(*n* = 45)	(*n* = 80)	(*n* = 33)
Too much	—	1 (2%)	1 (1%)	—
Too little	2 (4%)	4 (9%)	3 (4%)	2 (6%)
About right	45 (96%)	40 (89%)	76 (95%)	31 (94%)
Information increased knowledge?	(*n* = 47)	(*n* = 45)	(*n* = 80)	(*n* = 33)
Strongly agree/agree	30 (64%)	27 (60%)	57 (71%)	28 (85%)
Neither	10 (21%)	13 (29%)	17 (21%)	4 (12%)
Disagree/strongly disagree	7 (15%)	5 (11%)	6 (8%)	1 (3%)
Information presented helped me decide whether to be screened or not?	(*n* = 47)	(*n* = 44)	(*n* = 80)	(*n* = 33)
Strongly agree/agree	34 (72%)	31 (71%)	56 (70%)	28 (76%)
Neither	7 (15%)	10 (23%)	17 (21%)	3 (9%)
Disagree/strongly disagree	6 (13%)	3 (7%)	7 (9%)	2 (6%)
Readiness to have test/discussion?	(*n* = 47)	(*n* = 44)	(*n* = 80)	(*n* = 33)
Not ready	8 (17%)	6 (14%)	19 (24%)	54 (15%)
Ready	38 (81%)	29 (66%)	57 (71%)	28 (85%)
Unsure	1 (2%)	9 (21%)	4 (5%)	—

*Question not asked of first 18 participants.
